# Combining deep learning and crowd-sourcing images to predict housing quality in rural China

**DOI:** 10.1038/s41598-022-23679-8

**Published:** 2022-11-15

**Authors:** Weipan Xu, Yu Gu, Yifan Chen, Yongtian Wang, Luan Chen, Weihuan Deng, Xun Li

**Affiliations:** 1 Department of Urban and Regional Planning, School of Geography and Planning, Sun Yat-Sen University, Guangzhou, China; 2 Augur Intelligence Technology Co., Ltd., Guangzhou, China

**Keywords:** Computational science, Computer science

## Abstract

Housing quality is essential to human well-being, security and health. Monitoring the housing quality is crucial for unveiling the socioeconomic development status and providing political proposals. However, depicting the nationwide housing quality in large-scale and fine detail is exceedingly rare in remote rural areas owing to the high cost of canonical survey methods. Taking rural China as an example, we collect massive rural house images for housing quality assessment by various volunteers and further build up a deep learning model based on the assessed images to realize an automatic prediction for huge raw house images. As a result, the model performance achieves a high *R*^2^ of 0.76. Afterward, the housing qualities of 10,000 Chinese villages are estimated based on 50,000 unlabeled geo-images, and an apparent spatial heterogeneity is discovered. Specifically, divided by Qinling Mountains-Huaihe River Line, housing quality in southern China is much better than in northern China. Our method provides high-resolution predictions of housing quality across the extensive rural area, which could be a complementary tool for automatical monitoring of housing change and supporting house-related policymaking.

## Introduction

Humans carry out many daily activities in their houses; therefore, the housing quality determines their living quality and well-being. Currently, massive numbers of people face a severe housing problem worldwide, especially in the rural area. In the United States, more than 6.7 million rural households live in houses lacking necessary domestic facilities while where they spend over 30% of their income^1^; in China, although dwelling conditions have improved significantly since 1978^2,3^, hundred million villagers are eagerly looking forward to high-quality housing^4,5^. Following the Sustainable Development Goal proposal, the accessibility of safe and affordable houses has been a significant in eliminating poverty and promoting human well-being^6^.

In rural China, a house not only decides residential quality but also becomes a vessel containing a lifetime of labor achievement of rural residents^7^. Moreover, this vessel possesses a time-honored history that considers the house representative of family, wealth and even marriage^8–11^. Hence, rural housing quality is the barometer of rural wealth status^12^. Consequently, depicting the distribution of housing quality across the rural area in finer detail and further unveiling its spatial pattern is of remarkable significance for understanding villagers’ lives situations and exploring the association between housing quality and rural wealth.

In the past, research on housing quality evaluation in rural areas mainly relies on questionnaires and field investigation data, like Demographic and Health Surveys^13^ and the China General Social Survey^14^. For instance, when leveraging the house area per capita data from China Household Finance Survey to evaluate the housing quality, Wang finds that the inequality of house assets in rural China is much greater than in urban area^12^. Similarly, housing size, construction materials and domestic facilities are also adopted to assess housing quality and analyze the socio-economic development in rural areas^15–17^. Nevertheless, these traditional data collection approaches require resources. They are costly, inefficient, and limited to capturing well-rounded and large-scale housing quality data to showcase a nationwide spatial distribution for holistic spatial pattern unveiling.

Recently, rapidly developed house-observed imagery data provide a promising opportunity for identifying the housing quality at a large scale and with high efficiency. As the most prevailing ground-observed data, satellite imagery is utilized broadly to extract various external elements of individual houses, i.e., the building footprint^18^, new-old building classification^19^ and construction material^20^. Still, owing to the vertical angle of satellite sensors, more detailed housing features are inevitably ignored, which are crucial to determining housing quality^21^.

Instead, house-observed imagery by cell phones or camera provides detailed and intuitive features based on which humans are able to evaluate the housing quality. From the house appearance, people can judge the building height or size, construction materials, decoration styles, facilities like air conditioners, and even some safety information like wall cracks and so on, and further conclude the housing quality. For example, street view imagery prevails in understanding the urban physical construction environment and human habitat perception^22,23^. This data records abundant and various housing features from the pedestrian perspective, and are used to extract not only the physical objectives like housing height, density, and greening level^24–27^, but also indicators of subjective cognition of habitat comfortability, building style and street quality via computer vision and AI techniques^28–31^. All in all, street view imagery is viable for housing quality assessment of individual house automatically and at a large scale.

Nevertheless, data scarcity seriously hinders the application of housing quality evaluation in rural areas. Urban street view imagery depends on either big technology companies like Google Maps, or the crowd-sourcing platform of Mapillary and some social media platform of Twitter and Sina Weibo^32–34^. Such pay more attention to cities instead of under-developed rural areas. Thus, there are almost no accessible house-observed images of the village to determine rural housing quality.

**Figure 1:**
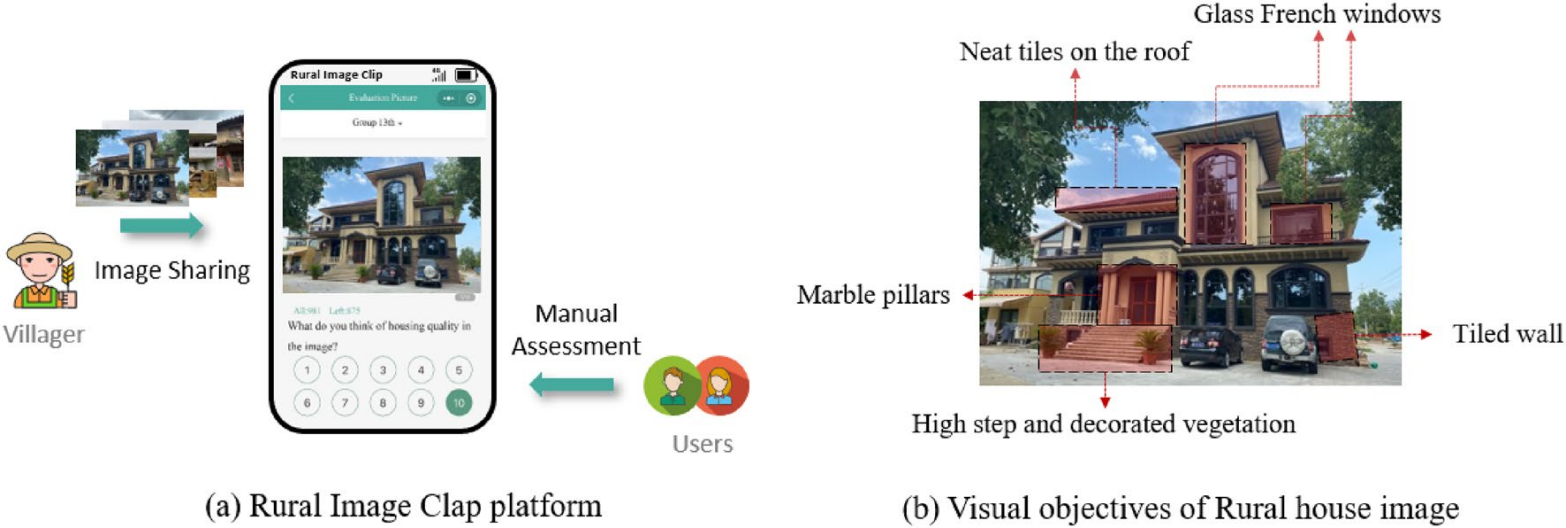
Crowed-sourcing rural image sharing and assessment platform. The photographs are provided by Guangzhou Urban Rural Habitat Planning and Design Limited.

To fill this gap, we establish a crowed-sourcing platform called *Rural Image Clap,* as Fig. 1a demonstrates. It allows villagers to share their surrounding rural images on their mobile phones, including houses, farmland, ponds, gardens, roads, etc. Meanwhile, other users can assess the housing quality manually according to these rural house images. Figure 1b exemplifies a typical rural house of high quality with luxurious external decoration, clean and broad gardens and a vast residential area. There is an emerging opportunity to use large-scale and massive rural house images to evaluate housing quality throughout Chinese villages. Benefiting from the crowdsourcing platform, we collected 15,699 rural house images of 83 counties in 27 province-level administrative regions to train the housing quality prediction model.

**Figure 2:**
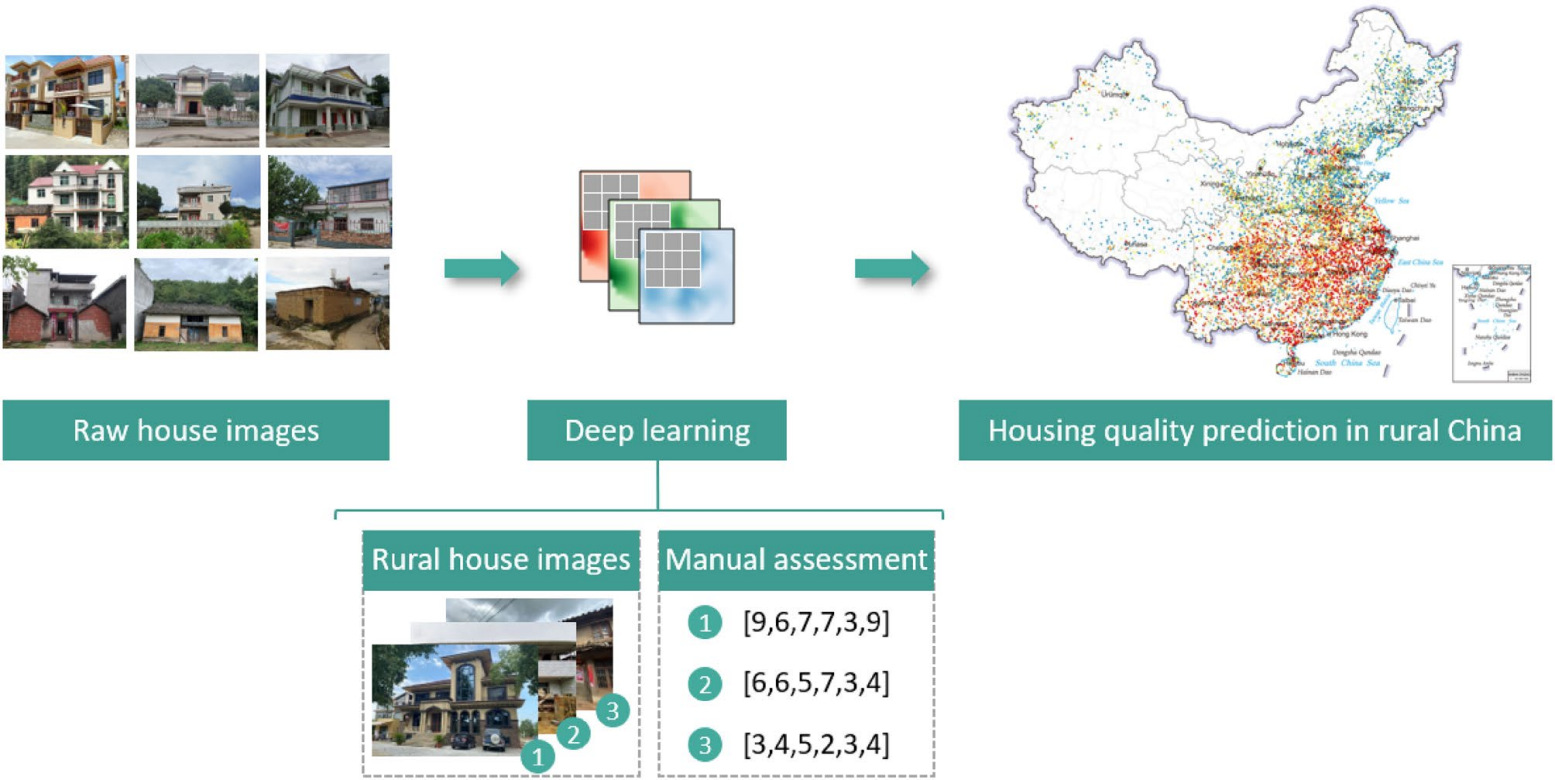
A framework for predicting rural housing quality by combing deep learning and crowd-sourcing images. The photographs are provided by Guangzhou Urban Rural Habitat Planning and Design Limited.

This paper proposes a framework that evaluates rural housing quality by combining deep learning and crowd-sourcing images. Figure 2 shows that sizeable rural house images and their manual quality assessment are available to train a deep learning model. Then raw-shared rural house images are added to the well-trained model to predict their housing quality automatically and precisely. Later, we can unravel the spatial distribution pattern of the rural house with different attributes in capacious and develop-unbalanced regions and offer a political suggestion for handling inequality of living conditions and promoting sustainable development in the rural area.

Based on the proposed framework, this study, for the first time, predicts the housing quality of individual house in the whole rural China. Specifically, there are two highlights: (a) fulfilling the striking gap of research data scarcity in rural areas through constructing a crowd-sourcing rural image-sharing platform; (b) proving the feasibility and effectiveness of combining rural-view images and deep learning to evaluate the housing quality, commonly regarded as a subjective and hard-assessed but exceedingly important indicators reflecting well-being and poverty elimination.

## Result

### Rural housing quality distribution in China

**Figure 3:**
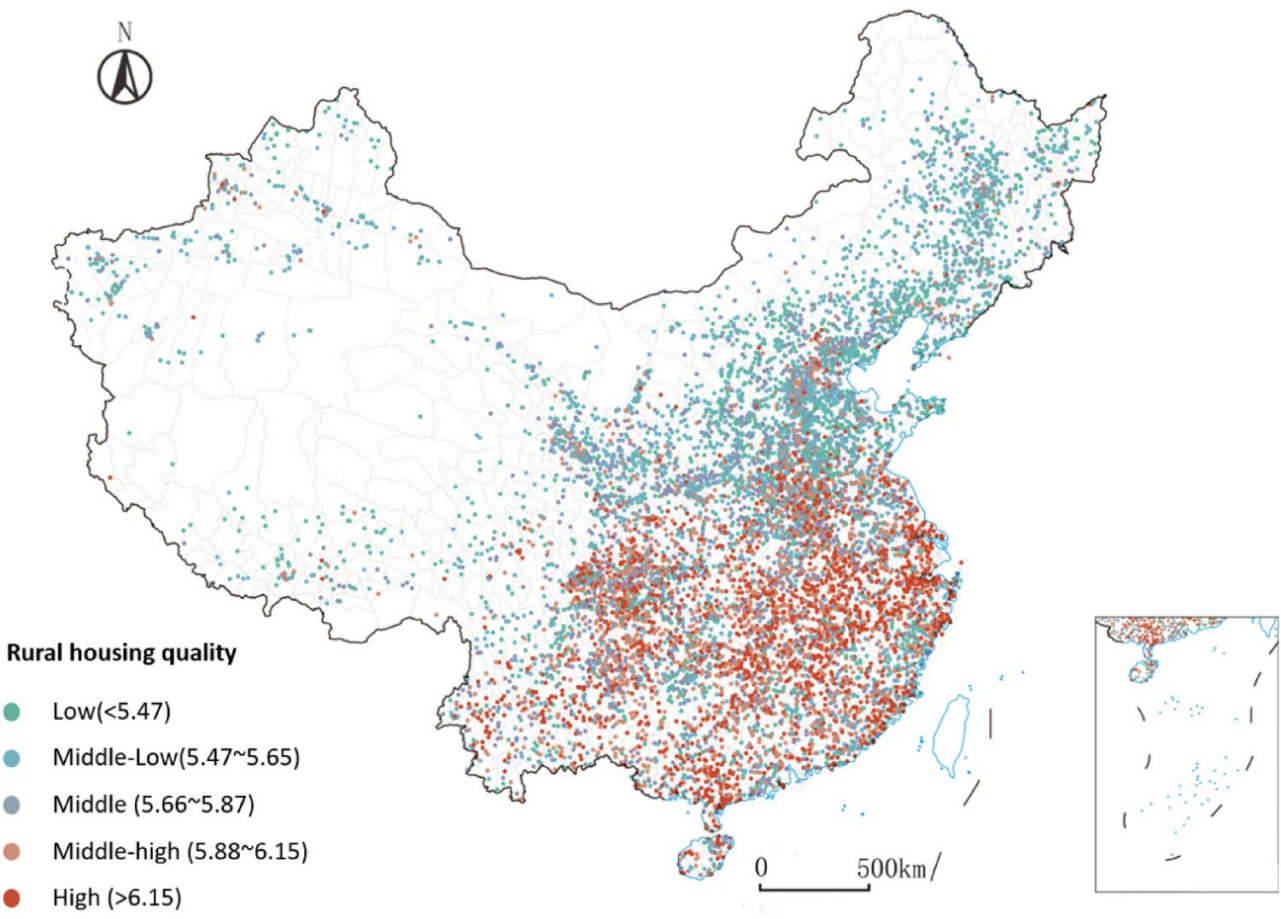
The distribution of predicted housing quality in rural China. Each dot represents one village. The map is created by Arcgis 10.0, and the base map is provided by Standard Map Service System (http://bzdt.ch.mnr.gov.cn).

Based on the well-trained deep learning of housing quality prediction (“Methods” section), another unlabeled 50,000 rural house images are evaluated and given their quality scores. These images cover 10,000 villages around China, so that a broader picture of rural housing quality is made (Fig. 3). In the image scale, the mean value of predicted housing quality is 5.81. There exists a significant difference among images, with a maximum value of 7.81 and a minimum value of 4.02. Image scores are aggregated at the village level by calculating the mean value of five images from each village. Thus, we can observe a massive inequality in housing quality across rural China (Fig. 3). Roughly, housing quality in southern villages is much higher than that in northern.

### Spatial pattern of housing quality in rural China in multi-scales

**Figure 4:**
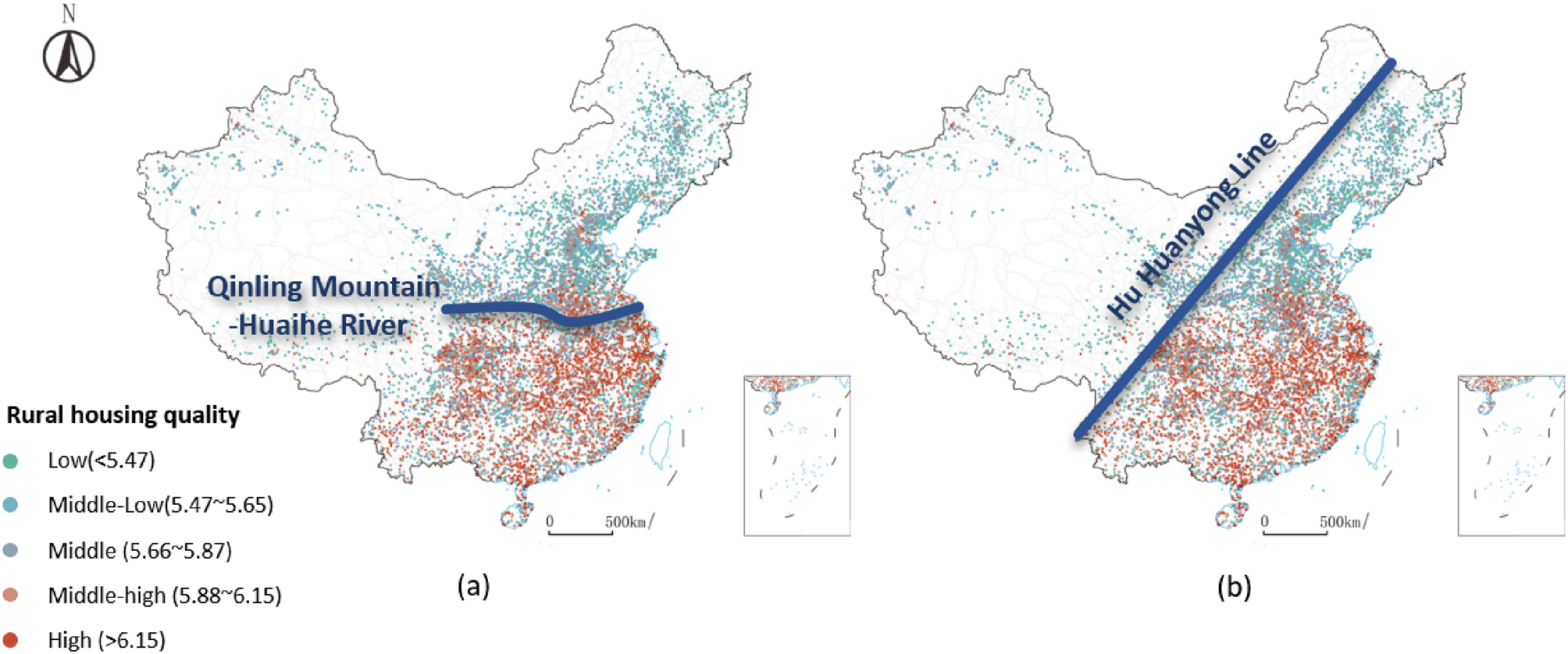
Two nationwide dividing lines for spatial pattern of housing quality in China. The map is created by Arcgis 10.0, and the base map is provided by Standard Map Service System (http://bzdt.ch.mnr.gov.cn).

Generally, a noticeable spatial variation in housing quality distribution could be noticed within southern and northern China. With the Qinling Mountain-Huaihe River as the dividing line between south and north, the rural housing quality in northern China ranges between middle and low. In contrast, the high-quality rural houses in the southern villages become intensive and dense (Fig. 4a). Specifically, the southern and northern villages’ average housing quality values are 5.96 and 5.61, respectively.

Additionally, when we plot the “Hu Huanyong Line” on the map, which is a significant proxy to differentiate the socio-economic development in China^35^, rural housing quality in the West is conspicuously lower compared with the East. Specifically, high and middle-high-quality villages are rare in east-south villages unless they junction but gather in south-eastern China (Fig. 4b). The average housing quality values of eastern and western villages are 5.80 and 5.56, respectively.

These two spatial patterns of rural housing quality distribution on a national scale positively correlate with the socio-economic development status. The vast economic imbalance in various areas is of significance in China. In the past, people usually paid great attention to the development imbalance between urban and rural areas instead of to the inherent difference between villages^36^. According to the predicted result, rural villagers in more affluent rural regions construct better-quality houses than under-developed areas.

**Figure 5:**
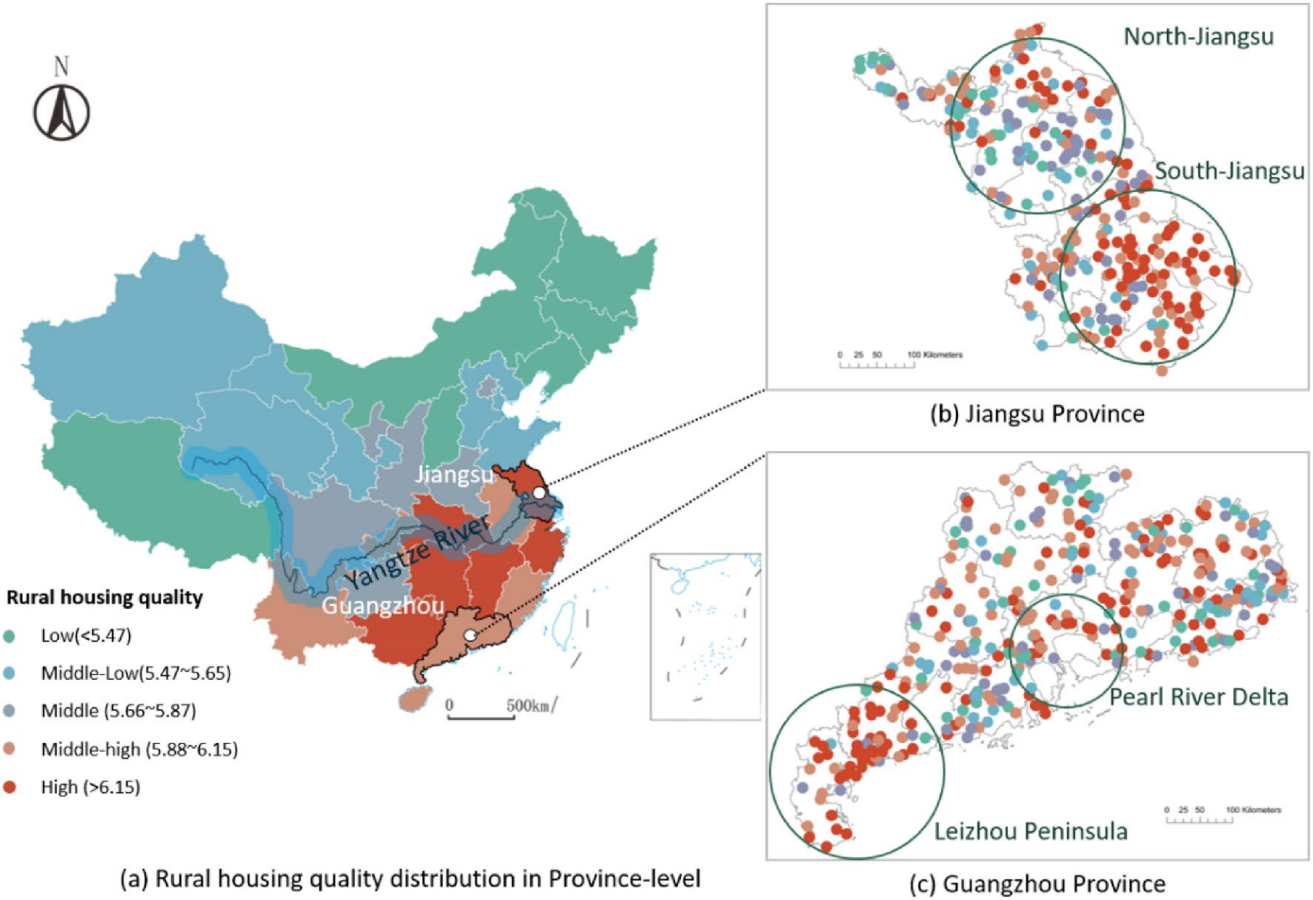
The distribution of rural housing quality on a regional scale. (**a**) Displays the housing quality at the province level, (**b**) looks at the Jiangsu province and (**c**) displays the housing quality at the village level. Each dot represents one village. The map is created by Arcgis 10.0, and the base map is provided by Standard Map Service System (http://bzdt.ch.mnr.gov.cn).

Furthermore, the rural housing quality is aggregated to the province scale as Fig. 5a shows. We can see that the rural housing quality of the province grows from edge provinces to provinces along the Yangtze River. It is worth noting that the pattern is partly different from the regional wealth level, such as the two most well-developed metropolitan areas, “Beijing-Tianjin-Hebei” and Pearl River Delta, did not construct high-quality villages like the Yangtze River Delta. Therefore, we do not think villagers in the more affluent province are expected to build higher-quality rural houses.

In a finer-granularity view, rural housing quality is affected by other factors, not only regional wealth. For example, in Guangdong province, the wealthiest area is the Pearl River Delta; whereas the high-quality rural houses mainly gather in Leizhou Peninsula, which belongs to the poor area (Fig. 5c). Some literature proposes an opinion that the Cantonese have a tradition of building well-decorated and tall houses in their hometown because they commonly have a hometown complex^37^ and are eager to attain an identity and respect for their household wealth from neighbors and relatives^38^.

On the contrary, the rural houses in southern Jiangsu province, a famous wealthy district, have a corresponding higher quality compared with a relatively poor area of northern Jiangsu (Fig. 5b). Consequently, even though the rural house is the paramount representative of wealth, other impact factors like culture, convention, and territoriality also constantly affect the spatial pattern of rural housing quality distribution.

### Indication for rural socio-economic development level

**Figure 6:**
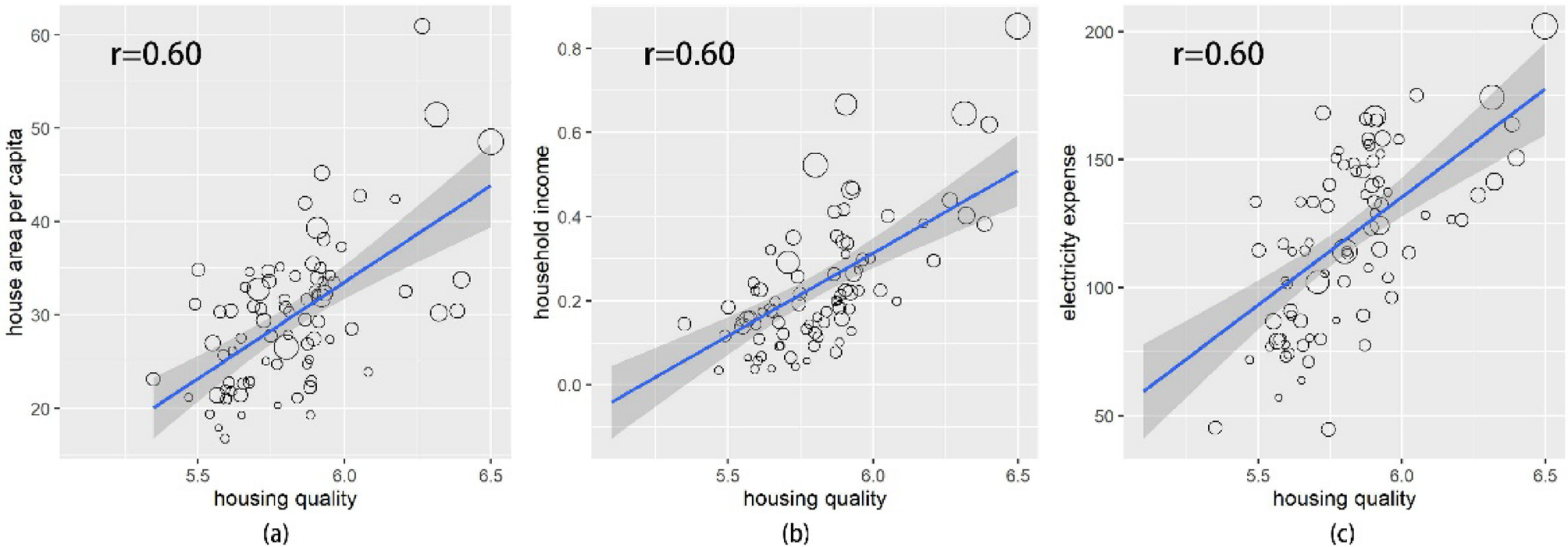
The relationship between housing quality and socio-economic indexes. Housing quality is the average predicted housing quality of one county. The dot size represents the disposal income per capita.

Can housing quality be a feasible indicator of rural socio-economic status? Here three indexes, *housing area per capita*, *household income* and *electric expense* are used to reflect the average household wealth (see “Methods” section). A house is the most critical asset that rural families own. *Housing area per capita* could be a good indicator for stock assets, while *household income* and *electric expense* could represent the flow of wealth. It is found that housing quality is highly positively correlated with *housing area per capita*, *household income* and *electric expense,* whose Pearson’s coefficients all reach a high level of 0.60 (Fig. 6). Households with houses of higher quality are more likely to have a prominent place, a higher income and more electricity. When people earn much money, they are more eager to invest on the house assets, construct a more comfortable and beautiful house, and buy more electric house appliances. Hence, housing quality can well reflect the rural socio-economic development level.

## Discussion

For this paper, we collected thousands of rural house images from a crowd-sourcing platform *Rural Image Clap*. Then we used deep learning to predict the housing quality in rural China with these images. According to the expected result, we depicted a nationwide map of rural housing distribution and further unveiled its spatial pattern on multiple scales.

As previous research mentioned, rural housing quality is primarily decided upon by the local wealth and socio-economic development level based on either little-sample questionnaires, field surveying or coarse-granularity general statistics. Thus lacking holistic and elaborated cognition. We discovered that rural housing quality conforms with the regional wealth on the whole through the nationwide predicted result. Nevertheless, other factors affect how much incomes villagers invest in constructing houses, like the culture of an area. This achievement enhances our understanding of the status of villagers’ living conditions in capacious rural China, which complements the research gap in rural housing quality.

Besides, mapping and monitoring rural housing quality can have good policy implications. Housing quality is highly related to one’s wealthy, so that it can help policy-makers tell the impoverished villages from the rich ones, and further carry out some precise aid to the impoverished villages, even poor household. Roughly, it is found that villages in southern, eastern China are much richer than those in northern and western China. To narrow the regional economic gap, more efficient policies should be made for the northern villages. For example, China has launched a “toilet revolution” in the rural area, especially in the northern area, where the house toilet is often a dry pail latrine without water to wash, and apart from the main house. The housing quality estimation can help to evaluate which house needs further policy aid.

Finally, there are still some limitations in this study. Firstly, the rural image assessment wholly depends on subjective perception from users all over the country; hence, if the south users occupy the majority, the north rustic house may be under-estimated compared with southern dwellings with the same investment, and vice versa. The bias of crowd-sourcing images likely affects the predicted reliability. Additionally, deep learning is a black-box model with which we do not figure out why it derives the housing quality instead of an end-to-end mapping between images and quality scores. In the future, we aim to discover the interpretability of deep learning for housing quality prediction to ensure which features contribute to the appropriate housing scores.

## Methods

### Datasets

This paper includes two primary image datasets, of which one is labeled while the other is not. The deep learning model is trained based on the labelled ones, while the unlabeled ones are used to map the inequality map of housing quality around rural China.

**Figure 7:**
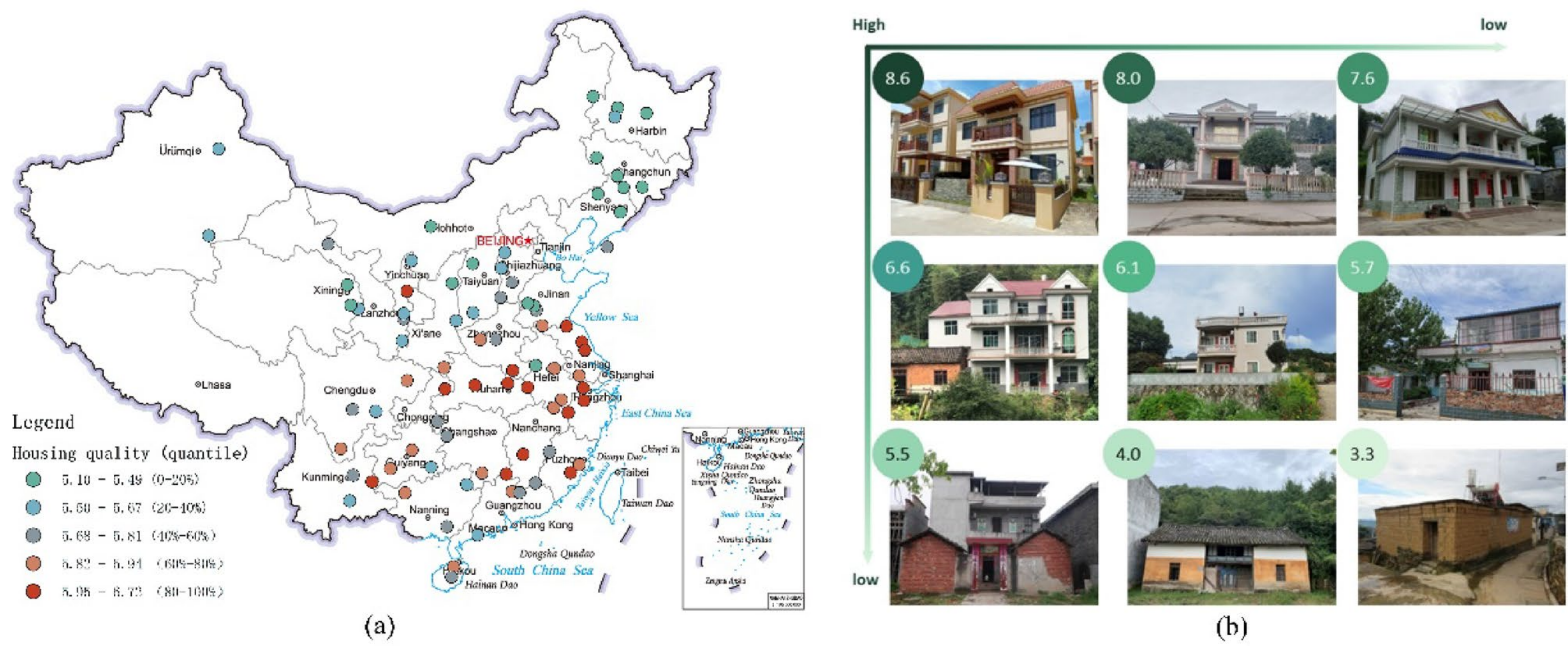
User’s assessment for crowd-scouring rural house images. (**a**) Presents the average housing quality of 83 counties across China with a warmer dot meaning higher quality, and (**b**) displays nine different levels of housing quality from high to low. The map is created by Arcgis 10.0, and the base map is provided by Standard Map Service System (http://bzdt.ch.mnr.gov.cn). The photographs are provided by Guangzhou Guangzhou Urban Rural Habitat Planning and Design Limited.

In detail, the labeled image dataset is collected from a crowdsourcing platform called *Rural Image Clap.* Totally, 15,699 rural house images are selected, which cover 83 counties of 27 province-level administrative regions (Fig. 7). These counties launched *Rural Construction Estimation* activations that investigate the problems and shortcomings of the rural area. Investigators shared their pastoral images from mobile phones, so that we could access them.

### Crowdsourcing assessment on housing quality

TO grade the housing quality from images, we advocate a scoring activity on the crowdsourcing platform. Around China, 350 users join in. Each image is scored 22.6 times on average, 15 times at the very least and 38 times at most. Users give each image a comprehensive score, from the worst score of 1 to the best 10. On the scoring platform, we also provide some tips for judging housing quality. Specifically, we suggest looking at (1) Whether the exterior wall of the house is secure and beautiful; (2) Whether the roof, doors, and windows are beautiful or have a unique local style; (3) Whether the house contains balconies, steps, canopies, ramps, chimneys and other auxiliary parts; (4) Whether the house has air conditioning, parking spots, or other facilities; (5) Whether users strongly desire to live in it. Therefore, we can guarantee a reasonable assessment to the greatest possible extent.

Due to the multiple assessments from different users, each image has at least 15 quality score. To calculate a unique value of housing quality for per image, firstly, the quality assessed score set of the image $i$ is denoted as ${Q}_{i}=[{s}_{i}^{1},{s}_{i}^{2},\dots , {s}_{i}^{n}]$, where $n$ indicates that there are $n$ users who give quality scores to $i$. Then, ${Q}_{i}$ is transformed to ${Q}_{i}^{^{\prime}}$ by Eq. (1), where ${q}_{i}^{j}$ is the proportion of score $j$($j$ ∈ [1, 10]) in all scores. Last, ${Q}_{i}^{^{\prime}}$ of each image is calculated to a weighted average as the final unique value ${A}_{i}$ by Eq. (2).

1 $${Q}_{i}^{^{\prime}}=\left[{q}_{i}^{1},{q}_{i}^{2},\dots ,{q}_{i}^{10}\right],$$

2 $${A}_{i}={\sum }_{j\in \left[\mathrm{1,10}\right]}{q}_{i}^{j}\times j\left({q}_{i}^{j}\in {Q}_{i}^{^{\prime}}\right).$$

The average score for all assessed 15,699 rural house images is 5.7, while the highest and lowest scores are 8.7 and 3.2, respectively. Besides, the whole housing quality scores follow a normal distribution with a standard deviation of 0.85. Taking 9 rural house images as a typical example, as Fig. 7 displayed, the rural houses with a high floor, luxuriant decoration, and wall-embraced gardens are seen as high quality. On the contrary, the low-quality houses represent a primitive, fragile and unsafe sense without the ability to resist storm. They rarely possess external leisure space like a garden but directly face the roads. After scoring, we get a labeled image dataset for training housing quality prediction model.

Another image dataset is unlabeled, and used to map the inequality of housing quality around China. This dataset contains 50,000 rural house images of 10,000 villages from 2452 counties of 30 province-level administrative regions (Fig. 3). Each village has 5 images. This dataset provides a broader picture of housing quality across rural China.

Finally, in the result section, some socio-economic indexes are collected from the survey. During *Rural Construction Estimation*, 134,461 household questionnaires were collected, based on which two indexes, *Household Income* and *Electricity Expense* are calculated. *Household Income* is the proportion of households with annual income above 60,000 Yuan in a specific county. *Electricity Expense* is the average household’s expenditure on monthly electricity for a particular county. The third index, *house area per capita* is collected from the census.

### Deep neural network

A bulk of rural house images and partial manual assessment of housing quality from users provide a viable opportunity to use automatically Deep Learning to predict housing quality all over rural China.

In brief, Deep Learning could extract the high-dimensional feature from input images using a deep neural network and construct the mapping between features and housing quality using a full-connected layer. Thus, effectively extracting features from images determines the predicting performance. Among various deep learning models like AlexNet^39^, VGG^40^, and ResNet^41^, DenseNet^42^ gets a striking success in image processing tasks owing to its robust feature extraction ability which is thus adopted in rural housing quality prediction throughout this paper.

**Figure 8:**
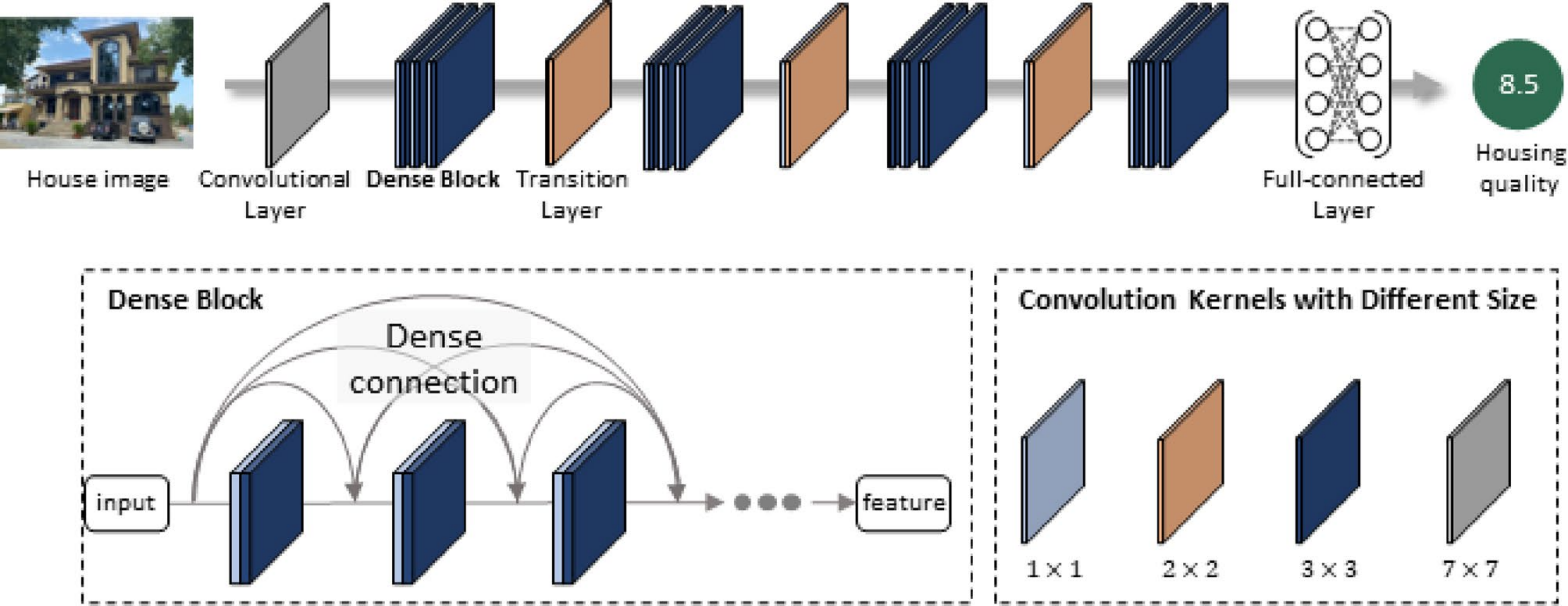
The architecture of DenseNet.

The architecture of DenseNet is shown in Fig. 8. It consists of a single convolutional layer, four Dense Blocks, three Transition layers and one full-connected layer in sequence. Specifically, as Fig. 8 shows, the Dense Block comprises several modules with two convolutional kernels of varying sizes. Furthermore, these modules are connected by “Dense Connection”, which entirely takes advantage of shallow convolution features to enhance model performance. Otherwise, the Transition layer connects the adjacent Dense Blocks to deliver the extracted features^42^. At last, constantly convolved high-dimensional features are input to a fully-connected layer to predict the housing quality.

### Model training and evaluation

For Deep Neural Network, the predicted accuracy is determined by the model’s parameters. Therefore, to obtain the optimal value of parameters, the assessed house images and their quality scores are used to train the model to update parameters, namely Back Propagation until the loss function achieves convergence. As mentioned in experimental data, each rural house image receives multiple scores. In model prediction, we expect the model could learn the score distribution instead of a simple unique value, that considers the subjective assessment bias for different users. Consequently, the input and output of the model is the relative frequency of each score $j$($j\in [\mathrm{1,10}]$) in all scores.

On this basis, the loss function of Mean Square Error (MSE) is used to measure the deviation between predicted and true house quality in each iteration, which is formulated at Eq. (3).

3 $$MSE=\frac{\sum_{i\in [1,n]}{(\sum_{j\in \left[\mathrm{1,10}\right]}{q}_{i}^{j}\times j- \sum_{j\in \left[\mathrm{1,10}\right]}{\widehat{q}}_{i}^{j}\times j)}^{2}}{n},$$

where n is the number of rural images, ${q}_{i}^{j}$ and ${\widehat{q}}_{i}^{j}$ ($j\in [\mathrm{1,10}]$)is respectively the true and predicted relative frequency of each quality score for rural house image $i$ ($i\in [1,n]$).

After model training, the performance of the trained DenseNet is evaluated byMSE of Eq. (3) and ${R}^{2}$ formulated at Eq. (4). Most importantly, although the formula of MSE in this section is the same as model training, the former one is used to represent the model prediction accuracy, while the rear one is an indicator for updating model parameters until convergence in training.

4 $${R}^{2}=1-\frac{\sum_{i=1}^{n}({\widehat{{A}_{i}}-{A}_{i})}^{2}}{\sum_{i=1}^{n}({\overline{Ai }-{A}_{i})}^{2}} ,$$

where, $\widehat{{A}_{i}}$ and ${A}_{i}$ are the predicted and true unique value of housing quality of images $i$ respectively; $\overline{Ai }$ is the average of $\widehat{{A}_{i}}$.

In detail, ${R}^{2}$ can examine the fitting degree between dependent and independent variables of the model, where the result of 1 demonstrates a perfect fit, and it means a reliable model for predictions.

### Experimental set and environment

The 15,699 images with users’ assessment are divided into the training, validation and test sets with the proportion of 80%, 20%, and 10% respectively. The hyper-parameters of the DenseNet model are set as below: batchsize is 32, epochs are 100, and the learning rate is initially 1 × 10^–5^ and adaptively adjusts with the decreasing degree of 0.1.

To further illustrate the model effectiveness of the proposed framework, other two Deep Learning models are adopted in the same dataset and set. For the deep learning, the model performance among different models is mainly due to their convolution backbone. Before DenseNet, the most prevailing model in high-dimensional image feature extraction is the VGG^40^ and ResNet^41^, in specific, the backbone of VGG is multiple successive convolutional blocks with a linear connection that achieve a good performance in image recognition task, while ResNet propose a shortcut connection to further solve the deep degradation problem hence adopted broadly as a basic CNN model. In addition to the two classical model, Dense improve the shortcut to a dense connection, as described in the before-mentioned section.

All model training is carried out in a PC with a CPU of Intel(R) Core (TM) i7-10700 K CPU with 3.80 GHz, and a GPU of NVIDIA GeForce RTX 3060.

### Model performance

Leveraging 15,699 rural housing images and their manual quality assessment, the DenseNet for housing quality prediction is trained, as well as the comparative models of VGG and ResNet. Their model performances are listed in Table 1.

**Table 1:** The model performance of VGG, ResNet, and DenseNet models.

Model	R^2^	$\mathrm{MSE}$
VGG16	0.6027	0.2717
ResNet50	0.5285	0.2912
DenseNet	0.7598	0.1636

It can be seen that the DenseNet has the best prediction accuracy of an R^2^ of 0.7598 and an MSE of 0.1636, respectively, which is better than VGG16 and ResNet50, illustrating the proposed framework is well competent in extracting features from rural house images to predict the housing quality.

Furthermore, specific raw rural house images predicted by the DenseNet are presented in Table 2. When observing these images in order of scores from low to high, it could be noticed that predicted quality scores were generally precisely consistent with their visual information in general. For instance, the rural house of 7.5 scores is much better than 6.0, likewise for house images of 6.0 and 4.5. On the other hand, comparing the images of 7.7 and 7.5, it is hard to assert that the former house is absolutely better than the latter one, because their housing features have their own advantages in decoration, style, and usage area. As such, this model has a sufficient capacity to evaluate a rough quality score over 1 or 2 scores for a rural house image, but limited in predicting relatively precise scores within 0.5 score. This result proves that the model is effective for the task in this study, because even human experts could not give an undisputed assessment of two similar rural house images owing to objective aesthetics and habitat environment difference.

**Table 2:** Raw rural house images and their predicted scores of housing quality.

Image	Quality score	Image	Quality score	Image	Quality score
	4.0		4.5		5.0
	5.5		6.0		6.5
	7.0		7.5		7.7

## Data Availability

The dataset and code for training models during the current study are available in the github repository https://github.com/Tutu-wq/housingquality.git.
